# Implementation Intentions Related to Self-Regulatory Processes Do Not Enhance Learning in a Multimedia Environment

**DOI:** 10.3389/fpsyg.2020.00046

**Published:** 2020-01-22

**Authors:** Emely Hoch, Katharina Scheiter, Anne Schüler

**Affiliations:** ^1^Multiple Representations Lab, Leibniz-Institut für Wissensmedien, Tübingen, Germany; ^2^LEAD Graduate School and Research Network, University of Tübingen, Tübingen, Germany

**Keywords:** multimedia learning, self-regulated learning, implementation intentions, cognitive strategies, self-efficacy beliefs, effort

## Abstract

Learners face various obstacles during learning from illustrated texts that can be conceptualized against the backdrop of frameworks of self-regulated learning. According to these frameworks, for learning to be successful, students must use appropriate cognitive strategies, hold adequate self-efficacy beliefs, and invest sufficient effort in learning. We investigated whether implementation intentions (if-then-plans) relating to these self-regulatory processes improve learning in a multimedia environment and whether they differ in their effectiveness depending on the self-regulatory process that they address. Learners were either asked to internalize an implementation intention relating to cognitive strategies, self-efficacy beliefs, or effort, or they did not receive any instructional support (control condition). Then, they learned about a mechanical system from a multimedia message and finally were tested on the learned contents. Contrary to expectations, none of the implementation intentions increased learning outcome, compared with the control condition, nor did the conditions differ from each other. However, implementation intentions interacted with the self-efficacy beliefs that learners already held. Higher self-efficacy beliefs were associated with better learning outcome, unless learners received an implementation intention telling them to use a multimedia-specific cognitive strategy. Interfering cognitive processes are discussed as a possible explanation for this interaction. In summary, implementation intentions should be further investigated and optimized before they can be implemented in real-life learning contexts.

## Introduction

Multimedia materials (i.e., illustrated texts) are commonly used in school textbooks and other educational media. Even though such formats have been shown to enhance learning (e.g., Butcher, 2014), they also impose challenges on learners (e.g., Scheiter et al., 2017). These challenges are related to cognitive, motivational, and behavioral aspects of students’ processing and hence predict difficulties regarding their self-regulated learning from multimedia (cf. Pintrich, 2000). Self-regulated learning refers to how learners manage their own learning, that is, how they direct their thoughts, feelings, and actions toward achieving a goal (Zimmerman, 2000). More specifically, Pintrich (2000) described self-regulated learning as an “active, constructive process, whereby learners set goals for their learning and then attempt to monitor, regulate, and control their cognition, motivation, and behavior, guided and constrained by their goals and the contextual features in the environment” (p. 453). Self-regulation in learning is important as it has been shown that students’ use of self-regulated learning strategies is strongly associated with superior academic functioning (Zimmerman and Martinez-Pons, 1986). Based on an analysis of these self-regulatory challenges against the backdrop of the literature on multimedia learning in relation to Pintrich’s self-regulated learning framework, in the present study, it is investigated whether supporting learners to overcome these self-regulatory challenges via implementation intentions (if-then plans, Gollwitzer, 1999) will foster learning from multimedia. Implementation intentions are a self-regulatory strategy that helps to translate any kind of plan into action and, thus, can be adjusted to the various challenges in multimedia learning.

### Self-Regulatory Challenges in Multimedia Learning

Multimedia learning requires learners to cognitively engage in learning, to have confidence in their own learning capabilities, and to allocate sufficient effort to learning to maximally profit from multimedia representations (Moreno, 2006; Mayer, 2014b). Hereinafter, these challenges of multimedia learning will be elaborated and put into context with models of self-regulated learning.

For successful multimedia learning, learners must cognitively process text and picture information and relate both types of information with each other to build one coherent mental representation (Schnotz, 2014; Mayer, 2014a). There is plenty of evidence that multimedia material is often processed insufficiently when presented without instructional support (e.g., Scheiter et al., 2017). For instance, learners make only few attempts to connect text and picture information (e.g., Ainsworth et al., 2002; Schwonke et al., 2009). However, building interconnections between text and picture information is necessary in order to really profit from multimedia material (Seufert, 2003; Mason et al., 2013; Mayer, 2014a). Accordingly, several interventions that aimed at guiding learners toward building referential connections were found to improve learning (e.g., Scheiter and Eitel, 2015; Stalbovs et al., 2015; Richter et al., 2016; Mason et al., 2017).

Besides, there is consistent evidence showing that processing multimedia material is demanding and that learners struggle to execute the required cognitive processes (Renkl and Scheiter, 2017). Reliance on text information (Hegarty and Just, 1993; Hannus and Hyönä, 1999; Schwonke et al., 2009) might be an indicator that learners feel overwhelmed by the multimedia presentation (Lowe, 1999, 2003) and thus prefer to rely on the more familiar representation for learning, namely text. This experience of being overwhelmed may be caused by a lack of perceived self-efficacy (Bandura and Locke, 2003), that is, a lack of confidence in one’s own abilities to cope with the learning task (Zimmerman, 1990). Self-efficacy is important as it positively relates to deeper elaborating the learning contents and to performance (Berger and Karabenick, 2011).

At the same time, the apparent simplicity of pictorial information and the appearance of multimedia as entertaining (Salomon, 1984) may lead learners to perceive multimedia material as being underwhelming (Lowe, 2003). In line with this, research has shown that learners tend to rely on a *multimedia heuristic* (Serra and Dunlosky, 2010) in that they associate multimedia learning material with better learning outcomes. Thus, they become overconfident in their own performance when learning with multimedia materials compared with learning from text alone (Ackerman and Leiser, 2014; Jaeger and Wiley, 2014; Eitel, 2016). Such overconfidence in monitoring one’s level of understanding is problematic because it is likely to affect regulation of subsequent learning (Bjork et al., 2013). In particular, learners may decide to invest only little effort and time, or even to stop learning prematurely (Son and Metcalfe, 2000; Dunlosky and Rawson, 2012). However, for multimedia learning to be effective, it is necessary to invest sufficient effort for actively engaging in cognitive processing (Mayer, 2014a,b). Both, a lack of self-efficacy beliefs and relying on the multimedia heuristic, are problems that relate to an inadequate evaluation of one’s own learning status, which is also called a lack of metacognitive accuracy (Nelson, 1996). Judging one’s own learning in a given task as accurately as possible is important, as it is assumed to determine subsequent learning behavior (Nelson et al., 1994; Thiede and Dunlosky, 1999; Bjork et al., 2013).

In short, learners must choose suitable cognitive strategies, hold adequate self-efficacy beliefs, and invest sufficient cognitive effort in learning to take full advantage of multimedia materials. These requirements for successful multimedia learning can be conceptualized against the backdrop of models of self-regulated learning. Such models mostly not only focus on the selection, combination, and coordination of cognitive strategies but also include motivational and behavioral aspects (Corno, 1986; Zimmerman, 1990; Boekaerts, 1999; Pintrich, 2000; Veenman, 2011b). Pintrich (2000) developed a framework that describes four areas of self-regulated learning: cognition, motivation, behavior, and context. *Cognition* describes the different cognitive strategies that learners know about and that they use. Control and regulation of cognition, that is, the selection and use of cognitive strategies, are designated to be the central aspect in self-regulated learning. Thus, making only few interconnections between text and picture information can be classified as a lack of cognitive regulation in multimedia learning. *Motivation* refers to the learners’ self-efficacy beliefs and values, interest, and liking of the task. Self-efficacy beliefs are the confidence in one’s own capability to achieve the desired outcomes (Zimmerman, 2000). This motivational level might be problematic in learners who feel overwhelmed by the multimedia materials. Motivational control and regulation of one’s self-efficacy beliefs might be obtained by positive self-talk (“I can do it!”). *Behavior* relates to the general effort that learners invest in the learning task. Behavioral control and regulation include managing time and effort according to task requirements. An adequate behavioral regulation is jeopardized when learners feel underwhelmed by the materials and, thus, only invest little effort in learning. *Context* refers to the external environment and circumstances of the learning task.

To conclude, learners face different challenges in multimedia learning that can be classified as occurring at a cognitive, a motivational, or a behavioral level of self-regulated learning. Accordingly, a generic intervention that can be modified flexibly to match any of the problems, namely implementation intentions, was used in our experiment to support cognitive, motivational, and behavioral self-regulation in multimedia learning.

### Using Implementation Intentions to Foster Learning

Implementation intentions specifically address self-regulation (Gollwitzer, 1999). These are if-then action plans connecting a favorable situation (if) with a goal-directed behavioral response (then), specifying when, where, and how the behavior should be executed (e.g., “*If* I am in situation X, *then* I will perform goal directed behavior Y.”). Thus, implementation intentions closely relate to production rules (e.g., ACT-R: Anderson, 1996; *WWW&H*-rule: Veenman et al., 2006). In self-regulated learning research, production rules specify declarative (what to do) and procedural (when to do it) information, which are both important for the successful use of (cognitive) strategies. Implementation intentions have been shown to be effective to support goal-oriented behavior (*d* = 0.65 in a meta-analysis from Gollwitzer and Sheeran, 2006), which is explained by their cognitive efficiency. Subjects who internalized implementation intentions had better access to memory representations of the situation specified in the if-part (Aarts et al., 1999; Parks-Stamm et al., 2007; Webb and Sheeran, 2007, 2008). Furthermore, implementation intentions were found to lead to automatic activation of the behavior specified in the then-part (Gollwitzer and Brandstätter, 1997; Bayer et al., 2009), which makes cognitively demanding control of behavior dispensable. The high accessibility of the situation together with the automaticity of the behavior makes implementation intentions very efficient in terms of cognitive resources (Brandstätter et al., 2001). Furthermore, it was found that strong goal commitment (Sheeran et al., 2005; De Nooijer et al., 2006), high personal interest in the relevant goal (Koestner et al., 2002), and specificity of the plans (De Vet et al., 2011) increased an effect of implementation intentions. Other research has shown that implementation intentions are particularly effective when they are applied to self-regulatory problems. This was investigated with individuals that basically struggle in self-regulation such as drug addicts under withdrawal, schizophrenic patients, or children with ADHD (Brandstätter et al., 2001; Gawrilow and Gollwitzer, 2008; Gawrilow et al., 2011). Furthermore, implementation intentions are very flexible regarding their content. Implementation intentions have already been used to effectively support self-regulation in educational contexts. Stalbovs et al. (2015) successfully used implementation intentions to improve cognition in self-regulated learning. Before studying a multimedia message, learners were instructed with implementation intentions that comprised multimedia-specific cognitive strategies (e.g., “If I have read a paragraph, then I will search the picture for the contents described therein.”) and were then tested on the just-studied contents. Implementation intentions improved participants’ learning outcomes compared with a control group that did not receive any instructional support.

Bayer and Gollwitzer (2007) studied the effectiveness of an implementation intention relating to learners’ motivational level of self-regulated learning. The implementation intention aimed at fostering self-efficacy beliefs through positive self-talk in a math test (“And if I start a new problem, then I will tell myself: I can solve it!”). Learners with the implementation intention solved more math tasks correctly than the control group who was not instructed via implementation intentions. However, experimentally induced self-efficacy was found to moderate the effect of implementation intentions (Wieber et al., 2010). Participants were initially given an easy or difficult task to induce high or low self-efficacy, respectively. Then, they were asked to solve analytic reasoning tasks of varying complexity and were given an implementation intention to evaluate the task solution (“And if I have found an initial solution, then I will double check it!”). Whereas one would assume that implementation intentions could compensate for low self-efficacy, no effect of implementation intentions was found when self-efficacy was low. Instead, results suggested that implementation intentions were effective only when self-efficacy was high, and items of the reasoning task were complex. However, the implementation intention referred to a strategy for solving the reasoning task (“double check it”). It did not refer to strengthening self-efficacy beliefs. Thus, the implementation intention did not aim at regulating the self-regulatory challenge (overcoming low self-efficacy) caused by the self-efficacy manipulation.

The behavioral level of self-regulated learning was investigated in a study by Duckworth et al. (2011). Implementation intentions aimed to increase the time and effort that students invested in preparation for an exam several months later. Participants who formed implementation intentions, which were directed toward completing all practice tests in a supplied workbook, completed more tasks in the workbook than a control group.

Taken together, implementation intentions seem to be a helpful tool to support self-regulatory processes. The goal of the present study was to address the relative effectiveness of implementation intentions related to the three areas of self-regulated learning, which tackle problems that students may face in multimedia learning.

### Overview of Study and Hypotheses

The present study consisted of three different implementation intention conditions and a control condition. The implementation intentions related to the areas of Pintrich’s self-regulated learning framework. In the three experimental conditions, prior to learning, participants were instructed via an implementation intention to either make use of a multimedia-specific cognitive strategy (cognition), to increase their self-efficacy (motivation), or to increase the effort they invested in learning (behavior). Students in the control condition did not receive any support prior to learning. After studying the multimedia message, learning outcome was measured.

It was hypothesized that implementation intentions support self-regulatory processes of multimedia learning and thus improve learning outcome. Therefore, it was expected that all groups with implementation intentions outperformed the control group (H1a – learning outcome: control group < implementation intention groups). Moreover, the cognitive implementation intention was expected to be more effective than that related to motivation or behavior (H1b – learning outcome: motivation/behavior < cognition), as Pintrich assumes cognitive self-regulation as the central aspect of self-regulated learning. Furthermore, the cognitive implementation intention relates more specifically and directly to cognitive mechanisms, whereby specificity is known to increase the effect of implementation intentions (de Vet et al., 2011). Since both motivational and behavioral regulations are less specific aspects of self-regulated learning, no differences in learning outcome were expected between these groups. Taken together, the main hypothesis was that learning outcome should increase from control group to the motivation and the behavior group and that the cognition group should achieve the highest learning outcome (H1 – learning outcome: control group < motivation/behavior < cognition).

Beyond learning outcome, judgments of learning were assessed to test for metacognitive accuracy during learning, which was calculated as the judgments of learners in relation to their actual learning performance. Metacognitive accuracy should determine which self-regulatory problem, that is, over- or underestimation of learning performance, would be more pronounced. Implementation intentions relating to motivation and behavior both addressed issues, which are grounded in an inadequate metacognitive accuracy. Motivational problems refer to learners feeling overwhelmed by multimedia materials, which might lead them to underestimate their learning performance. Behavioral problems refer to learners feeling underwhelmed by multimedia materials, which might lead them to overestimate their learning performance. Thus, implementation intentions that foster motivation were assumed to boost self-efficacy and increase the judgments of learning compared to the control group (H2a). Implementation intentions relating to behavior were assumed to increase the effort in learning, reduce the influence of a multimedia heuristic, and thus decrease the judgments of learning compared to the control group (H2b). This increase in effort and temporal engagement should also be reflected in an increase in learning time for the behavior group (H3).

Additionally, learner characteristics were analyzed exploratively for their possible interactions with implementation intention effects on learning outcome. Considering these as possible moderators is important as implementation intentions were shown to be particularly effective when they tackle self-regulatory problems (e.g., Brandstätter et al., 2001; Gawrilow and Gollwitzer, 2008; Gawrilow et al., 2011). In particular, we assessed knowledge about multimedia-specific strategies, self-efficacy beliefs, and planned effort as they refer to the areas of self-regulated learning that were addressed by the implementation intentions. One might expect that implementation intentions would compensate for low levels of these learner characteristics, whereby learners having problems with cognition, motivation, and/or behavior should only benefit from the implementation intention relating to the respective problem they have. On the other hand, learners with high levels of these characteristics might not have problems in the specified areas of self-regulated learning, which is why they might not profit from an intervention. This assumption is related to the so-called expertise reversal effect (Kalyuga et al., 2003). This effect describes that instructional support can be highly effective for learners that have low expertise but is not effective or even negatively affects learners with high levels of expertise.

## Method

### Participants and Design

Students with a major in physics or musicology were not allowed to participate due to content-related closeness to the learning material (mechanical explanation of a musical instrument). One participant studying physics as well as two participants who did not follow instructions were excluded from analysis, which left a sample of 119 undergraduate students from a German university (102 female; *M*_age_ = 22.72 years, *SD* = 2.94). We performed a *post hoc* power analysis (G*Power: Erdfelder et al., 1996) based on an alpha-level of *α* = 0.05, our sample size of *N* = 119, and the effect size of *f* = 0.325 (in the meta-analysis reported as *d* = 0.65; Gollwitzer and Sheeran, 2006). The statistical power was 0.845. Participants received 10€ for participation or course credits. They were randomly assigned to one of four conditions: three experimental conditions who received an implementation intention relating to either cognition, motivation, or behavior, or a control group with no instructional support via implementation intentions.

### Instructional Materials

**Figure 1:**
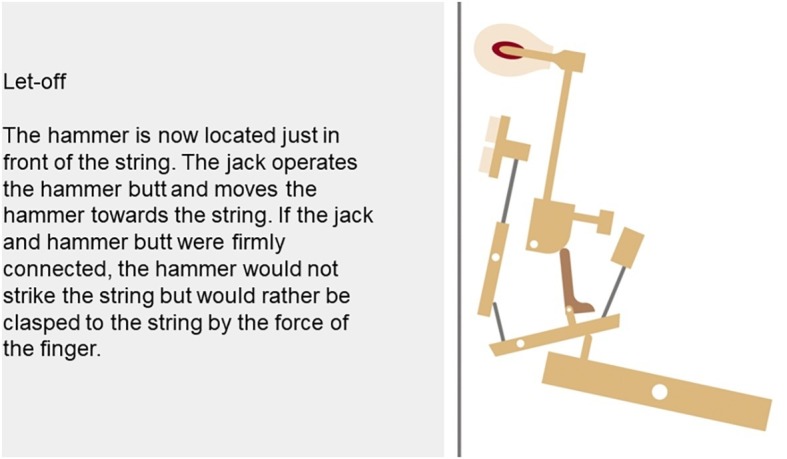
Screenshot from one page of the multimedia learning material (a German version was used in the experiment).

The functionality of a piano mechanism was used as the learning content (Figure 1). Participants were taught what happens inside a piano, how a tone is produced when a key of the piano is pressed, and how the components return to their initial positions when the key is released. The multimedia material was presented in nine pages, each containing text and a corresponding picture. Text and picture information were complementary. The pictures in the form of an animation as well as a verbal comprehension test and the open recall question have already been used in other studies (e.g., Boucheix and Lowe, 2010; Lowe and Boucheix, 2011, 2016).

### Measures

#### Learner Characteristics

Self-efficacy beliefs, planned effort, and knowledge about multimedia strategies were assessed to ensure that groups did not differ on those characteristics before manipulation. Participants had to rate their self-efficacy beliefs (11 items, e.g., “I think I am up to the difficulty of this task,” Cronbach’s *α* = 0.876) and the effort they planned to invest in the learning task (4 items, e.g., “I am really going to try as hard as I can on this task,” Cronbach’s *α* = 0.955) both on a scale from (1) *disagree* to (7) *agree*. The items were adopted from validated questionnaires but were adapted to fit the university context (short scale for measuring general self-efficacy beliefs/Allgemeine Selbstwirksamkeit Kurzskala = ASKU: Beierlein et al., 2013; Program for International Student Assessment = PISA: Kunter et al., 2002; Questionnaire on Current Motivation/Fragebogen zur Erfassung aktueller Motivation = QCM/FAM: Vollmeyer and Rheinberg, 2006). No selection was made, but all the items to measure self-efficacy beliefs and planned effort from the referenced questionnaires were used^1^. Means were calculated separately for self-efficacy beliefs and planned effort. Knowledge about multimedia strategies was assessed with a list of 24 expedient and inexpedient strategies in multimedia learning (e.g., “I look at the picture to check my understanding of the text”), from which participants chose which strategies they normally use when learning with multimedia (adapted from Scheiter et al., 2015). Only strategies that address integration of text and picture information were analyzed, as it is assumed an expedient and central process in multimedia learning (Mayer, 2014a). The number of expedient cognitive strategies that were ticked off was added up for each participant with a maximum of six expedient strategies.

#### Learning Outcome

Verbal comprehension, open recall, and pictorial recall were assessed to test for learning performance. The verbal comprehension test contained 23 verification items (max. 23 points; 1 point for each correct item), which referred to either the configuration of the piano elements (7 items) or the local kinematics of the system (16 items; e.g., “When the key is pressed, the whippen presses the damper on the string,” false). For each statement, students judged whether it was true or false. The open recall question asked for the overall functional mental model of the piano (“What happens to all elements of the piano system when a person presses and then releases the key? Please answer as accurately as possible.”). Points were given for every correctly remembered aspect (max. 24.5 points), whereas points were deduced for wrong descriptions. No points were given or deduced for omissions. In the pictorial recall test (max. 23.5 points), participants had to name elements, draw missing parts of the system, and sort pictures of the mechanism in the correct order. Open recall and pictorial recall items from 20% of the participants were coded by two independent raters with good interrater reliability (Cohen’s *κ* = 0.854). As the maximum scores as well as the pattern of results were similar for the three tests and their scores were highly correlated, learning outcome was computed across all tests and translated into percentage of total score (Cronbach’s *α* = 0.882).

#### Metacognitive Accuracy

Participants had to judge their learning in terms of their expected percentage correct on an upcoming test (judgment of learning, from 0 to 100) and to later judge their performance in the actually taken test (judgment of performance, from 0 to 100). The accuracy was calculated as the discrepancy between the participants’ judgments and their actual learning outcome as an indicator of metacognitive accuracy (Nelson, 1996; De Bruin and van Gog, 2012). Therefore, 0 points indicate perfect estimation, positive values show an overestimation, and negative values show an underestimation of one’s learning. Accuracy of participants’ judgments of performance was calculated as discrepancy only from the proportional scoring in the verbal comprehension test (corrected for guessing probability) because judgments of performance only referred to this test.

### Procedure

Participants were tested in groups of up to seven individuals working at separate workspaces in sessions lasting about 75 min. They first answered questions on demographic data and filled in the questionnaires on learner characteristics. All participants were informed that during the experiment, they would first learn something and then be tested on the contents. Then, participants in the experimental groups were introduced to implementation intentions as a tool to reach a specific goal. They were asked to have the goal of making optimal use of the learning material with the help of the respective implementation intention. Next, they wrote down their group’s specific pre-phrased implementation intention three times with the assignment to imagine how they would implement the specified action during learning (see Table 1 for exact wording of implementation intentions). This procedure was adopted from previous studies (e.g., Stalbovs et al., 2015). All implementation intention groups used the same if-part (“If I start a new page …”), since an effect could then be traced back to the varying action in the then-part only. Participants in the control group proceeded to learning without any instruction on implementation intentions.

**Table 1:** Implementation intentions used for instruction in each experimental condition.

	*n*	Implementation intention
Cognition	30	If I start a new page, then I will search the picture for the contents described in the text.
Motivation	28	If I start a new page, then I will tell myself: I can learn it!
Behavior	31	If I start a new page, then I will particularly concentrate on the content presented.

*Control group (n = 30) did not get instructed with implementation intentions. The cognitive implementation intention was adapted from Stalbovs et al. (2015). The motivational implementation intention was adapted from Bayer and Gollwitzer (2007)*.

The learning material was presented on laptops, and participants could determine learning time on their own. After the study phase, participants judged their learning in terms of their expected performance on a test about the piano mechanism (judgment of learning). After answering the verification items, participants estimated their performance on this just taken test (judgment of performance). Then, participants worked on the open recall question and the pictorial recall test (both paper and pencil).

## Results

Data analysis was conducted using R version 3.3.2 (R Core Team, 2016).

### Learner Characteristics

Analyses of variance (ANOVAs) or Kruskal-Wallis tests (for those variables where the normal distribution assumption for ANOVAs was violated) were conducted to test whether groups differed in learner characteristics (see Table 2 for means and standard deviations). There were no significant differences between conditions in knowledge about multimedia strategies, *H*(3) = 4.62, *p* = 0.202; self-efficacy beliefs, *F*(3, 115) = 0.52, *p* = 0.670, $\eta_{p}^{2}$ = 0.01; or planned effort, *H*(3) = 3.85, *p* = 0.278.

**Table 2:** Means and standard deviations of learner characteristics and dependent measures for the control group and the implementation intention groups (II).

	Control group	II cognition	II motivation	II behavior
Knowledge about multimedia strategies (0–6)	4.13 (1.46)	4.00 (1.68)	4.79 (1.03)	4.26 (1.21)
Self-efficacy beliefs (1–7)	5.28 (0.89)	5.03 (0.90)	5.06 (0.84)	5.18 (0.72)
Planned effort (1–7)	6.14 (0.76)	5.43 (1.41)	5.97 (1.04)	5.92 (0.73)
Learning outcome (%)	52.44 (11.17)	51.13 (14.10)	52.99 (16.09)	51.09 (12.64)
Learning time (min)	6.88 (3.81)	7.33 (3.78)	7.23 (3.47)	5.87 (1.90)
Judgments of learning (0–100)	66.33 (21.41)	56.67 (22.64)	66.07 (26.71)	59.68 (23.02)
Accuracy JoL	13.89 (18.01)	5.54 (16.58)	13.08 (17.94)	8.59 (19.08)
Judgments of performance (0–100)	50.33 (22.51)	43.00 (24.66)	48.57 (28.64)	40.97 (24.95)
Accuracy JoP	25.41 (26.36)	15.46 (24.81)	20.31 (25.38)	9.97 (24.49)

*Accuracy of judgments of learning (JoL) and judgments of performance (JoP) is given as discrepancy between participants’ judgments and scored performance of the learning outcome, whereby 0 points indicate perfect estimation, positive values indicate overestimation, and negative values indicate underestimation. It should be noted that the accuracy for judgments of performance only relates to performance in the verbal comprehension test*.

### Dependent Measures

#### Main Effect of Condition

Means and standard deviations for the dependent variables are presented in Table 2. ANOVAs or Kruskal-Wallis test was conducted to test whether groups differed on the dependent measures. Contrary to our hypothesis, learning outcome did not differ between conditions, *F*(3, 115) = 0.15, *p* = 0.932, $\eta_{p}^{2}$ = 0.004. Thus, none of the implementation intention groups outperformed the control group (H1). Furthermore, there were no differences between conditions in learning time, *H*(3) = 3.02, *p* = 0.388; judgments of learning, *H*(3) = 4.77, *p* = 0.189; and judgments of performance, *H*(3) = 2.95, *p* = 0.399. Contradicting our expectations, learning time did not increase for the behavior group compared with the control group (H3). Furthermore, the motivation group and the behavior group did not differ from the control group regarding judgments of learning (H2a, H2b).

In general, participants tended to overestimate their learning, which might speak in favor of learners having problems on a behavioral level of self-regulation. This was reflected in the accuracy for judgments of learning being significantly larger than 0, *t*(118) = 6.18, *p* < 0.001. Accuracy for judgments of learning did not differ among conditions, *F*(3, 115) = 1.42, *p* = 0.240, $\eta_{p}^{2}$ = 0.04. In general, participants also tended to overestimate their performance, which was reflected in the accuracy for judgments of performance being significantly larger than 0, *t*(118) = 7.53, *p* < 0.001. Accuracy for judgments of performance did not differ among conditions, *F*(3, 115) = 2.08, *p* = 0.107, $\eta_{p}^{2}$ = 0.05.

#### Moderating Role of Learner Characteristics

Separate regression analyses were conducted to test whether each of the three learner characteristics would moderate a possible effect of condition on learning outcome. Regression models contained one of the learner characteristics, the implementation intention conditions, and the two-way interaction as predictors. Implementation intention conditions were dummy coded with the control group as the baseline category. The continuous learner characteristic variables were *z* standardized.

**Figure 2:**
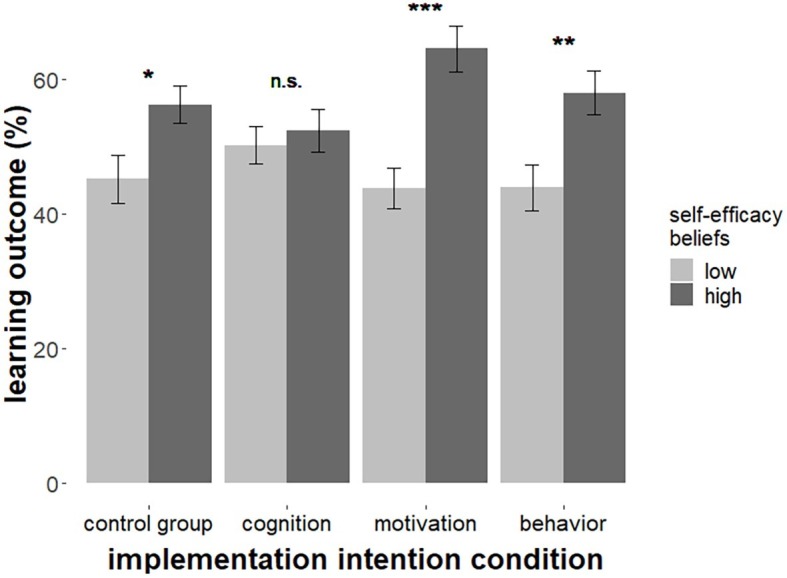
Learning outcome (percentage of correct answers) as a function of experimental condition and self-efficacy beliefs. Means at low and high levels of self-efficacy beliefs are estimated based on the simple slope analyses at −1 SD and +1 SD, respectively (^+^*p* ≤ 0.10, ^*^p ≤ 0.05, ^**^p ≤ 0.01, ^***^p ≤ 0.001).

Knowledge about multimedia strategies, *F*(1, 111) = 2.06, *p* = 0.154, $\eta_{p}^{2}$ = 0.02, and planned effort, *F*(1, 111) = 2.55, *p* = 0.113, $\eta_{p}^{2}$ = 0.02, was not predictive for learning outcome and did not significantly interact with implementation intention condition, both *F*s < 1. However, there was a positive effect of self-efficacy beliefs, *F*(1, 110) = 25.08, *p* < 0.001, $\eta_{p}^{2}$ = 0.19, and a significant interaction of self-efficacy beliefs with implementation intention condition, *F*(3, 110) = 3.23, *p* = 0.025, $\eta_{p}^{2}$ = 0.08 (data of one participant were excluded due to a Cook’s distance > 0.10, indicating an overly strong influence on the regression models outcome, Cook and Weisberg, 1980). The regression model (Table 3) explained about 20% of variance in the data, *F*(7, 110) = 5.12, *p* < 0.001, *R*^2^ = 0.197. To probe the interaction effect (Figure 2), a simple slope analysis for low (−1 SD) and high (+1 SD) values of self-efficacy beliefs was conducted (see Aiken and West, 1991). There were no differences between conditions for learners holding low self-efficacy beliefs, *F*(3, 110) = 1.07, *p* = 0.362, or high self-efficacy beliefs, *F*(3, 110) = 2.37, *p* = 0.075. We also tested the other possible perspective on the interaction to see whether implementation intentions differentially influenced the effect of self-efficacy beliefs on learning outcome. Simple slope analyses revealed that the relationship between self-efficacy beliefs and learning outcome differed between groups (Figure 2). There was a significant positive effect of self-efficacy beliefs in the control group, *B* = 5.52, *SE* = 2.26, *β* = 0.41, *p* = 0.016. This relationship was even stronger in the groups with behavioral implementation intentions, *B* = 7.03, *SE* = 2.51, *β* = 0.52, *p* = 0.006, and strongest in the group with motivational implementation intentions, *B* = 10.37, *SE* = 2.25, *β* = 0.77, *p* < 0.001. However, there was no relationship between self-efficacy beliefs and learning outcome in the group with cognitive implementation intentions, *B* = 1.09, *SE* = 2.04, *β* = 0.08, *p* = 0.594.

**Table 3:** Regression model to predict learning outcome (percentage of total score).

	*B*	SE_b_	*β*
Intercept	50.704	2.289	
Self-efficacy beliefs	5.517	2.255	0.411^*^
II behavior	0.195	3.146	0.016
II motivation	3.447	3.234	0.257
II cognition	0.581	3.184	0.490
II behavior × self-efficacy beliefs	1.514	3.374	0.113
II motivation × self-efficacy beliefs	4.849	3.186	0.409
II cognition × self-efficacy beliefs	−4.426	3.041	−0.330

*Model: F(7, 110) = 5.12, p < 0.001, R^2^ = 0.197*.

*Control group functions as baseline category. Implementation intention is abbreviated by “II”*.

+ p ≤ 0.10

* p ≤ 0.05;

** p ≤ 0.01;

*** *p ≤ 0.001*.

## Discussion

The aim of this study was to test the effects of implementation intentions on learning from multimedia. The different types of implementation intentions were designed to support areas of self-regulated learning that could be problematic in multimedia learning, namely cognition, motivation, and behavior. We expected implementation intentions to support self-regulatory processes of multimedia learning and thus to improve learning outcome compared to a control group that did not receive any instructional. In addition, the most specific implementation intention (relating to cognition) was expected to yield the highest test scores. Furthermore, we expected implementation intentions to affect learning time and judgments of learning.

Contrary to these assumptions, we did not find any effects of implementation intentions on learning outcome, learning time, or judgments of learning. However, implementation intentions interacted with learners’ pre-existing self-efficacy beliefs. Possible explanations for these findings will be discussed in the following.

At the core of this study is the argument that learners face various self-regulatory problems when learning with multimedia. Although there is a considerable amount of empirical evidence for these problems, it might be that in our experiment, there were no problems of self-regulation to begin with. However, given that learning outcome in the control group was merely around 52%, we believe that multimedia learning was challenging and that instructional support was indeed needed to improve learning.

Turning to the learners’ difficulties in multimedia learning, in the current study, we do not have explicit evidence regarding the relative relevance of the self-regulatory problems. The learner characteristic variables measuring knowledge about multimedia-specific strategies, self-efficacy beliefs, and planned effort all showed mean values above the scale midpoint. This suggests that learners reported to not facing any of the aforementioned problems in serious ways. However, self-reports are a subjective assessment and have been critiqued as being invalid indicators in the context of self-regulated learning (Veenman, 2011a,b). More objective indicators become available when looking at judgments of learning. Independent of condition, learners gave judgments that exceeded test performance, suggesting that they slightly overestimated their learning. This indicates that learners indeed might have had problems with the behavioral area of self-regulated learning. Based on the overestimation, one would expect implementation intentions relating to behavior to be helpful, since they require learners to invest more effort even when they feel (over-)confident in their performance. On the other hand, motivational implementation intentions should hinder learning since they would only increase overconfidence by boosting students’ self-efficacy beliefs. An issue to be resolved in future studies is to assess the specific self-regulatory problems that learners hold and then adapt implementation intentions accordingly. For instance, a specific sample with low levels for the learner characteristics could be selected to specifically test whether different results are then found for implementation intentions.

Another possible explanation for the lack of significant effects on the dependent variables relates to the particular if-part of the implementation intentions. The if-part was the same in all experimental conditions. The specified situation (“If I start a new page”) only occurred once on each page and in fact occurred before and not during processing the contents. Accordingly, the different actions specified in the then-part might have been triggered before but not during learning the content of a certain page. In addition, the low specificity of the implementation intentions might account for the missing implementation intention effect in our experiment compared with other studies (de Vet et al., 2011). We could not replicate the positive effect of the cognitive implementation intention from Stalbovs et al. (2015). However, Stalbovs et al. tied the cognitive strategies more specifically to the problematic situation (i.e., “If I have finished reading a page, then I will carefully re-read all paragraphs”). By contrast, the situation that we referred to in the implementation intentions did not explicitly address a particular self-regulatory challenge. However, part of the effectiveness of implementation intentions results from addressing specific and critical situations and ties them to helpful and goal-oriented behavior. It is possible that the critical situations differ for cognitive, motivational, and behavioral self-regulation, and hence, the implementation intentions need to be formulated in a more specific way.

Despite these unexpected results regarding the main absent effect of the implementation intentions, an interesting finding of the present study is the interaction between learners’ self-efficacy beliefs and the implementation intentions. We found a positive effect of self-efficacy beliefs on learning outcome for all learners that disappeared when they were instructed with a specific cognitive strategy. Berger and Karabenick (2011) explain the positive effect that results from high self-efficacy beliefs by assuming that high self-efficacy beliefs foster the use of more elaborated strategies. Instructing high self-efficacious learners in the cognition group with one multimedia-specific strategy then might have interfered with the more elaborated and adaptive strategies they might have used by themselves. Importantly, these findings regarding the moderating role of pre-existing self-efficacy beliefs need to be interpreted with caution as the analysis of the interaction was exploratory. Replication is needed before more definite conclusions can be drawn.

### Strengths and Limitations

We obtained sufficient statistical power (0.845) that could not explain for our non-significant results regarding the effectiveness of implementation intentions. A sensitivity power analysis with our sample size of 119 participants and power determined at 0.80 revealed that the size of the minimal detectable effect was *f* = 0.308, which is a medium or medium to large effect (Cohen, 1992). Thus, we cannot completely rule out that there was a small effect in our study; however, we can rule out that there was an effect of implementation intentions that is comparable in size to the one that was found in the meta-analysis (*f* = 0.325: Gollwitzer and Sheeran, 2006).

Another strength of the study lies in the fact that our hypotheses rest on a homogenous theoretical framework. We adapted Pintrich’s framework of self-regulated learning to multimedia learning scenarios and underpinned relevant aspects with findings from multimedia research. Even though this is a clear theory-driven approach, it is still difficult to derive effective interventions. Thus, the practical implementation of interventions that closely relate to theoretical conceptualizations remains an objective for future research.

This issue is also reflected in the formulation of the if-part of implementation intentions, which was identical across the experimental conditions. From an experimental perspective, keeping the situation constant and neutral while only varying the to-be-investigated dimension (the action in the then-part to support various areas of self-regulation) is a straightforward approach. However, this control constitutes a drawback as the relatively generic and neutral situational cue might not be suited best for supporting the different areas of self-regulated learning.

## Conclusions

Taken together, the results from the present experiment revealed that even though learners indicated that they were well equipped for multimedia learning, their performance was relatively poor. However, implementation intentions relating to cognitive, motivational, and behavioral self-regulatory problems did not improve learning outcome. Further research is needed to investigate the self-regulatory problems themselves, how implementation intentions can be adjusted to help learners to overcome these self-regulatory problems, and finally, how implementation intentions should be tailored to meet the learners’ individual characteristics.

## Data Availability Statement

The raw data supporting the conclusions of this article will be made available by the authors, without undue reservation, to any qualified researcher.

## Ethics Statement

The study protocol was reviewed and approved by the Leibniz-Institut für Wissensmedien (IWM) own ethics committee. All subjects gave written informed consent for participation.

## Author Contributions

All authors listed have made a substantial, direct and intellectual contribution to the work, and approved it for publication.

### Conflict of Interest

The authors declare that the research was conducted in the absence of any commercial or financial relationships that could be construed as a potential conflict of interest.

## Footnotes

^1^A confirmatory factor analysis for all items (self-efficacy beliefs and effort) showed that the two scales align with a two-factor solution; hence, it is possible to distinguish between items that measure self-efficacy beliefs and planned effort. The confirmatory factor analysis for planned effort resulted in good fit indices (CFI = 0.999, RMSEA = 0.034, SRMR = 0.007). However, the confirmatory factor analysis for self-efficacy beliefs resulted in poor fit indices (CFI = 0.756, RMSEA = 0.183, SRMR = 0.098). When excluding five items that measure self-efficacy beliefs with only low factor loading, fit indices improved for self-efficacy beliefs (CFI = 0.992, RMSEA = 0.053, SRMR = 0.027). With this reduced set of items, the same pattern of results was found. Thus, all items were kept for the analysis.
